# Controlled human malaria infections using aseptic, purified cryopreserved *Plasmodium falciparum* sporozoites administered by needle and syringe

**DOI:** 10.1186/1475-2875-13-S1-P12

**Published:** 2014-09-22

**Authors:** Peter Billingsley, B Kim Lee Sim, Else Bijker, Meta Roestenberg, Kirsten Lyke, Matthew Laurens, Benjamin Mordmueller, Patricia Gomez, Seif Shekalaghe, Susanne Hodgson, Adrian Hill, Elizabeth Juma, Bernhards Ogutu, Bertrand Lell, Pedro Alonso, Salim Abdullah, Peter Kremsner, Marcel Tanner, Robert Sauerwein, Stephen Hoffman

**Affiliations:** 1 Sanaria Inc., Rockville, MD, USA; 2 Radboud University Nijmegen Medical Centre, Nijmegen, The Netherlands; 3 University of Leiden, Leiden, The Netherlands; 4 Center for Vaccine Development, University of Maryland, Baltimore, USA; 5 Universität Tübingen, Tübingen, Germany; 6 Centre de Recerca en Salut Internacional de Barcelona Hospital, Barcelona, Spain; 7 Ifakara Health Institute, Bagamoyo, Tanzania; 8 University of Oxford, Oxford, UK; 9 Kenya Institute for Medical Research, Nairobi, Kenya; 10 CERMEL, Lambaréné, Gabon; 11 Barcelona Centre for International Health Research, Barcelona, Spain; 12 Swiss Tropical and Public Health Institute, Basel, Switzerland

## 

Controlled human malaria infection (CHMI) studies, in which healthy volunteers are infected with *Plasmodium falciparum* (Pf), until recently have been performed primarily by using Pf-infected *Anopheles stephensi* mosquitoes to bite human subjects. This approach has proven highly successful for studies of drugs and vaccines. However, its use has been limited to the few clinical centers, which have access to Pf-infected mosquitoes. Sanaria Inc. has established a new CHMI methodology whereby aseptic, purified, cryopreserved, infectious sporozoites (SPZ) of Pf are used to infect volunteers when administered by needle and syringe. This product is known as PfSPZ Challenge. Here we summarize the results of seven clinical trials in the Netherlands, UK, Tanzania, USA, Germany, Spain, and Kenya including 178 subjects. Four of the trials were done in countries where CHMI had never been done before. These trials assessed intradermal, intramuscular and intravenous administration of varying doses of PfSPZ Challenge. The three primary goals of 100% infection rates, a prepatent period comparable to the bite of five Pf-infected mosquitoes (11 to 11.5 days) and a dose response have been met by intravenous and intramuscular administration. PfSPZ Challenge opens up the potential for CHMI to be done in any research facility set up for clinical malaria studies (see presentation by Sheehy *et al.*), including sites to which transport of *A. stephensi* is difficult or impossible (i.e. any site in Africa). The results of all the studies will be presented.

